# Supplementary material for: Transcriptome Analysis of mfs2-Defective Penicillium digitatum Mutant to Reveal Importance of Pdmfs2 in Developing Fungal Prochloraz Resistance
Source: Microorganisms. 2024 Apr 28;12(5):888. doi: 10.3390/microorganisms12050888 (PMC11123787; doi:10.3390/microorganisms12050888)
Supplement: Supplementary file 1 [file microorganisms-12-00888-s001.zip › microorganisms-2939896-supplementary.pdf]

Transcriptome analysis of *mfs2*-defective *Penicillium digitatum* mutant to reveal importance of *Pdmfs2* in developing fungal prochloraz-resistance

Rongrong Cuan[1], Shaoting Liu[2], Chuanyou Zhou[1], Shengqiang Wang[1], Yongliang Zheng[3], Yongze Yuan[1]*

[1]Hubei Key Laboratory of Genetic Regulation and Integrative Biology, School of Life Sciences, Central China Normal University, Wuhan, 430079, China;

[2]School of Political and Law, Huanggang Normal University, Huanggang, 438000, China;

[3]Hubei Key Laboratory of Economic Forest Germplasm Improvement and Resources Comprehensive Utilization & Hubei Collaborative Innovation Center for the Characteristic Resources Exploitation of Dabie Mountains, Huanggang Normal University, Huanggang, 438000, China.

*Correspondence: yyz2007980080@ccnu.edu.cn

Supplementary Materials

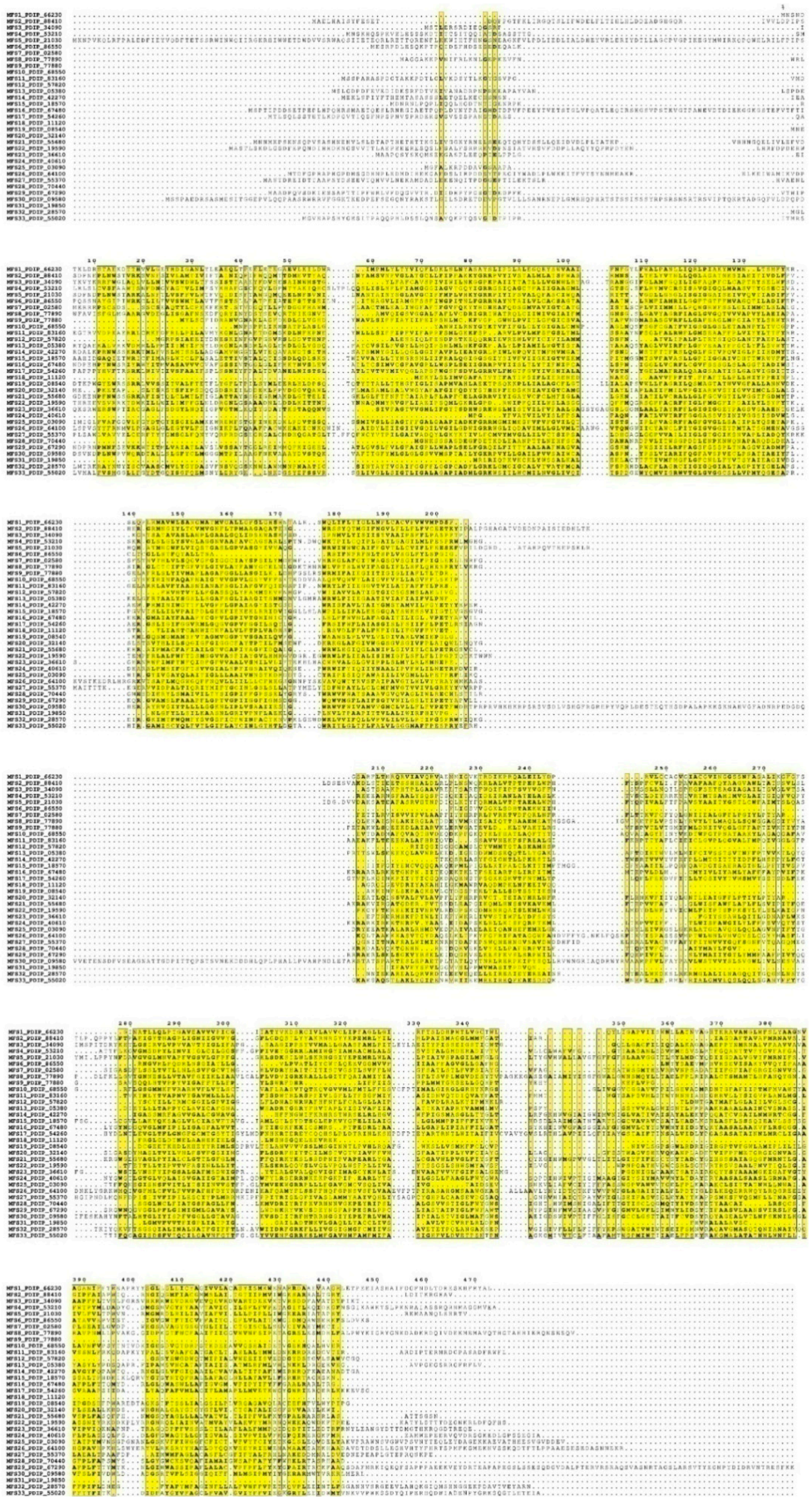

Figure S1. Protein homology alignments for multiple isoforms of MFS (MFS1-33), ABC (ABC1-8), and MATE (MATE1-3) in the present study.

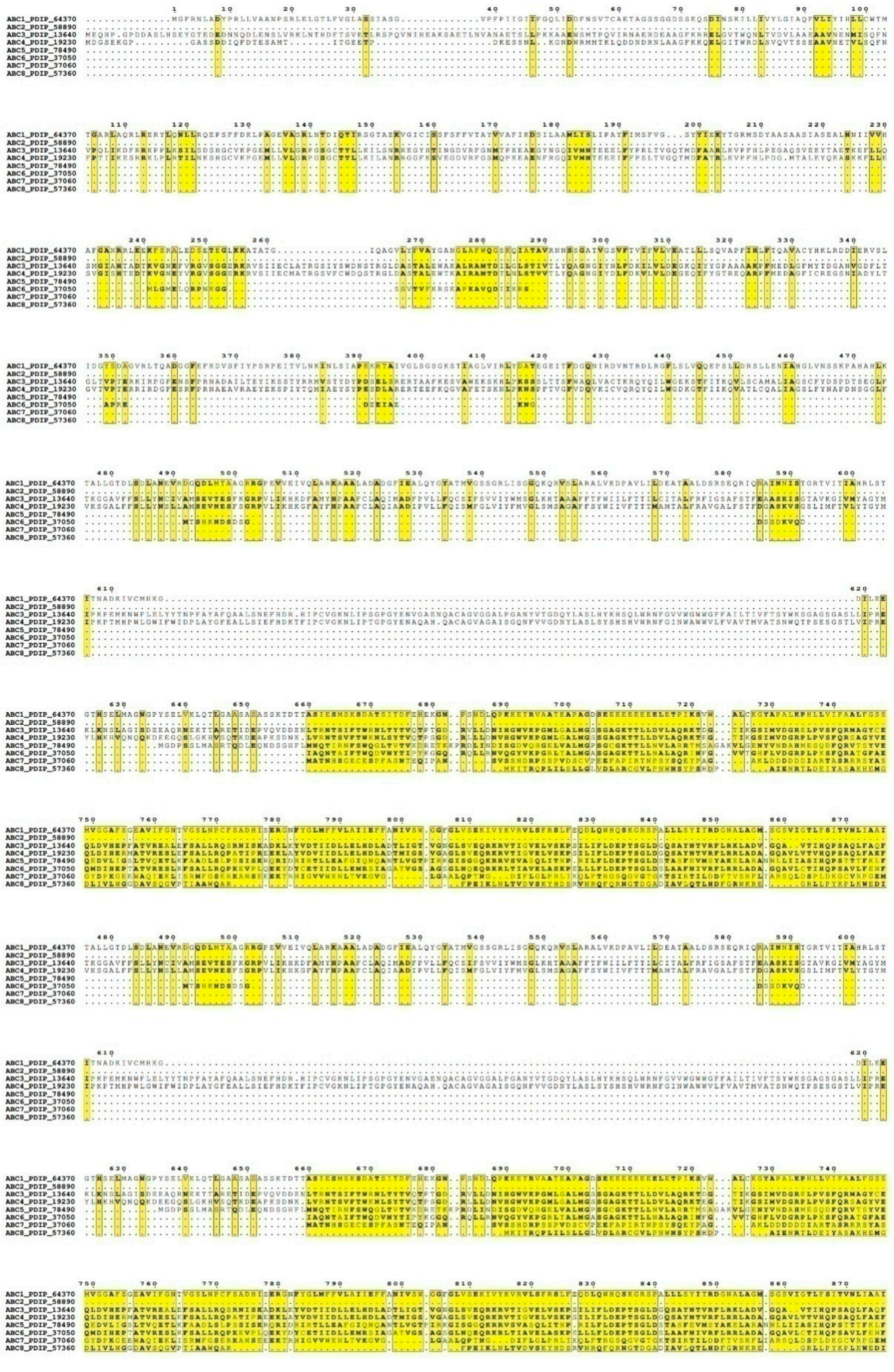

Figure S1 (continued). Protein homology alignments for multiple isoforms of MFS (MFS1-33), ABC (ABC1-8), and MATE (MATE1-3) in the present study.

```
                  1        10        20        30        40        50        60        70
MATE1_PDIP_56750  MANPDDARMGKILASSATGTSLLILIQLASRIFTFASNQLILRTLSPVVLGIAAQLELFQVSILYFSRES
MATE2_PDIP_40930  ......................................................................
MATE3_PDIP_05620  ......................................................................

                           80        90       100       110       120       130       140
MATE1_PDIP_56750  IRMAIQRQPIPFSPDNKKISLEADFEVTDSQSVASQAVVNVSYLSLALGIPASLMFTMLYQCLVPEEASN
MATE2_PDIP_40930  ......................................................................
MATE3_PDIP_05620  ......................................................................

                          150       160       170       180       190       200       210
MATE1_PDIP_56750  TAFFYHSVILIGASSLMELSIEPFFSVVQQHMLYEKRAAVEMPAAFLRSAVTSSAFICASQVNHDLGVLP
MATE2_PDIP_40930  .METYRDKGIMRAGTYVLLITSPLNAALNYVFCYT...................................
MATE3_PDIP_05620  ......................................................................

                          220       230       240       250       260       270       280
MATE1_PDIP_56750  FALGHLSYSLALICGYSLVLLRGTNTTRFSFSLTRIQSSTKNDTVTIYRNPSNYFLGRFSRELTSLAANV
MATE2_PDIP_40930  FEIGLLGAPLATGLCYWLSFALLVLYARFIRGSECWGGWSREAFQNLRTFARLALMGVVHVG.....TEW
MATE3_PDIP_05620  .................MGPTLGNNTAEVHAVTDLDDEHTHDSLLSLRWTVESGWWRRSS..........

                          290       300       310       320       330       340       350
MATE1_PDIP_56750  FFQSLVKHLLTQGDTMMLVALSGLEDQGIYSLASNYGGLIARIIFQPLEESSRNLFSALLSRGVDGKLKN
MATE2_PDIP_40930  WAFEIVALAAGRLGTVPLAAQS.........VVMTADQVLNTIPFGIGVATCGRVGNRLGKRDKTGAARA
MATE3_PDIP_05620  ...TLVKIWIANIDSTLVVTTD..........VYTYIGTVAEVLNECLPRAVWVTIADQVTRSYESRLEL

                          360       370       380       390       400       410       420
MATE1_PDIP_56750  VHIHTAKNHLVDILRAYQVMSILIFPLGPMMVPQLLHILGGRLWASPKTRDLLSVYCYYIPFLAFNGITE
MATE2_PDIP_40930  AHTSALLSVVFGGV....VLAVLMGTREQFAK............IFNDDASVVNLTAEVLPYVALFQIAD
MATE3_PDIP_05620  AHTLVAFQTVLGLI.....MSIILTSAAKAFT............ATFIPREARKASITYVQISAFSALSS

                          430       440       450       460       470       480       490
MATE1_PDIP_56750  AFVSSAANSQQIRKQTAWMGFFSACYALAAYMFLEVGNLGAYGLVLANIVNMVVRTFWSYSFIKSYLHRN
MATE2_PDIP_40930  GLNGSCGGSLRGMGRQHVG.........................AFVNLVSYYCGALPLGIWLAFHGRGL
MATE3_PDIP_05620  AIEVAVSNATRALDKPDVP.........................LVISSVKTVVNIILDLLIISEFHVGG

                          500       510         520       530
MATE1_PDIP_56750  GCSLYTKEVAIRPASFILSALA..SLFLGGFGLQLGIIKACVLSGSYALLM.
MATE2_PDIP_40930  KGLWVGQCTALYLVGALEWIIVARSQWDTEVRKAFQRMDVHEDLEDGAEEE.
MATE3_PDIP_05620  WSPNINMQAAIRLSCDMVAALTGLAYFIQTINLSEGRRSWHWIGNIPNFLPF
```

Figure S1 (continued). Protein homology alignments for multiple isoforms of MFS (MFS1-33), ABC (ABC1-8), and MATE (MATE1-3) in the present study.

Table S1 Primers used in the present qRT-PCR.

Gene name	Primer	Sequence (5’→3’)	Fragment size amplified
MFS1	F	GCCCACAGCACAAACCAAAG	108
	R	CATGCGCAAGGGTTGTTCAG	
MFS2	F	ACATCTTCCGGCGATTTCCAT	109
	R	GAACCTTAGCCCAAGTGCCA	
MFS3	F	CGATCATCGTACGTGAAACCC	106
	R	CTGCGACGGTTGACTGTTTG	
MFS4	F	AATGCTTCCTGAGTCTCCGC	107
	R	TGATTTCCTGCGAACACGGA	
MFS5	F	TGCATCCAGAGAGCATGACC	106
	R	ATCCCACAGCAAGAAGAGCC	
MFS6	F	CTGTGAGCTTTTGCAATGGGA	108
	R	CCCATTTGGTGATGGCAAGC	
MFS7	F	GCTAACACCAAGCACAGGGA	104
	R	CAGCCCTAGTGATGAGAACCA	
MFS8	F	CTTCACGGGCTTCGTCTACC	108
	R	TGGATCGCACATACCCATCG	
MFS9	F	CAAACTGGACAACGCTGACG	104
	R	GCAAGCTCACCTCGTCGATA	
MFS10	F	CGCTCCCCGCTTCATCTTAT	105
	R	GCCAGGTGTCCTTGACGTAG	
MFS11	F	GTGTGATGGACGAGGGGAC	110
	R	TGCAGACGTACACATCACCG	
MFS12	F	GGAGCTCTTATCACTGGGGG	107
	R	TGGATGCACAGCCCACTTAG	

MFS13	F	TAGCAACGCCAGCAGTATCT	105
	R	CTGGGATCCCCAGAAAGACA	
MFS14	F	ATCGGTTGGGTACTGTCGTG	102
	R	AATGGCTGAGACACAGGTCG	
MFS15	F	ATGCTAAACCGCCTCAACCA	106
	R	TCAGCGTGAAGAATGGCAGA	
MFS16	F	GGACCCTGTGTTCCCTGAAG	105
	R	TTCGTGGATGGAACAGAGCC	
MFS17	F	GTGAGCTCATGGCTTTCCGA	108
	R	ACGCACGTTAGGGTAAGACAC	
MFS18	F	TGGTTGGAGCGTTTGCTACT	106
	R	TGCCCCTGGAAACCAATCAA	
MFS19	F	AGCCATGGGGCTATGAATGG	106
	R	AGTGCTCGGGTGAATGACAC	
MFS20	F	GCTCCTTACGGCAAGGTGAA	104
	R	ACTGGTTCAAAAGCCACTGGA	
MFS21	F	GTCGGCGGTACTTTTGGAGA	105
	R	GTAGATGGGAGCACCGACTG	
MFS22	F	TGACCCGAATTCCATTGCCA	110
	R	CGTCCGGATCGAATCTGTGT	
MFS23	F	TCACTGTGTATCTTGGCCGC	103
	R	AAAGTGAGCACGTCAACGGA	
MFS24	F	CGAGTGTTGTCGGTATTGCAC	109
	R	TCTTTGCGGAGGAAAAGGCT	
MFS25	F	TGGGTAGGTCCCACTCACAT	103
	R	TCTTCATGGCGAGGATACCG	
MFS26	F	AGCAGTGGATCAACGCCATT	106
	R	ACGGACGGTAGAATTGCAGC	

MFS27	F	CCCAGAGCGCAGCTAATACT	109
	R	TCCTTCTCCAAATCGGCATCC	
MFS28	F	CTCACCTACTCCATTGGCCC	107
	R	CCCAAATTCCAGGCTAGGCA	
MFS29	F	TCGCGGTAAGACTTCGACTC	109
	R	CAGTGGAGCCCAGAGCAATG	
MFS30	F	GCGCCAAATCAACATTGGGA	108
	R	AGTCCCCGTTTCACCAAACA	
MFS31	F	CGTTTTCACATCGTGCCTGG	109
	R	CGTCCGGAGTTATACTCGCC	
MFS32	F	AAACACCCCAACTCGCCAAT	108
	R	AGACAGTGAGGAGAGGGAACT	
MFS33	F	CTCCTCGTATGCTCTACCGC	107
	R	GCAATGGCTGTAGAAACTGACA	
ABC1	F	AGGTGCCACTGTTGGATCTG	108
	R	GGGTAGCTGTCGGTGATGTT	
ABC2	F	GCGCTCTACAGAAGGTACGA	107
	R	GCAGAGGAGTTGCTTTTGGC	
ABC3	F	TCTGGTAAGCTGTCGTCTTCC	103
	R	AGCGGACCCGATAAATCGAA	
ABC4	F	CCTGGGCGTAAGTTTTCTTCA	110
	R	CTATTTGGGCGTAGGGGACC	
ABC5	F	TTCACCAAAGAGCGAGCCAA	106
	R	TGGCAGGAGATAGTGAGGACA	
ABC6	F	ATTTGCACGGTGTGAGCAAC	105
	R	GCGCAGCATAGAAGACACGA	
ABC7	F	GGTAATCAACGAGCGGGCTA	106
	R	AGAAACACACACCTTCAGACCT	

ABC8	F	AAACACGAGATGGGTGACCTC	108
	R	GCTTACAGCTTTTCAACCGTGT	
MATE1	F	ACTGGCACTTATCTGTGGCT	102
	R	GGCCAGTCTTACCTGGATTGT	
MATE2	F	GACGCGAGAACAATTTGCGA	105
	R	AGCCCATCGGCAATCTGAAA	
MATE3	F	GTGCAAGGCAAACGATGTGA	109
	R	AGTGCTTTACAACGCAGGGA	
1,3,6,8-Tetrahydroxynaphthalene reductase	F	TCCAAGCCGACCTGACAAAT	105
	R	TCTCAGTTCCTGCGTTCGAC	
4-Hydroxyphenylpyruvate dioxygenase	F	CAGTTGTCGACGAGGCATCT	111
	R	CAGTTGTCGACGAGGCATCT	
60S Acidic ribosomal phosphoprotein	F	CGAGAAGGTCATCTCCGAGC	108
	R	CAGCTATGGAAAGGGTCAGCA	
60S Ribosomal protein	F	TCCGCCGTCTGAGTTGTATG	106
	R	GACCTTAAGGGCTGCTCAGAA	
40S Ribosomal protein	F	CCGCAAGAAGATCCACGAGT	103
	R	ATCCGCTCTAAGGTGACGGA	
Ribosomal protein	F	CGAACAGTCAACTGGGGTGA	106
	R	TCTGGAAGCCGTTCTGGAAG	
Acetyl-CoA carboxylase	F	GGCTTATGAGACCTTCGGCA	104
	R	ATTGGTCGGCCATGCGAATA	
Alcohol dehydrogenase	F	GCGTCGGAGTACCACGTATT	109
	R	GGATGAGGCTCGTGTAGTCG	
Aldehyde dehydrogenase	F	TGGTTAGTCGTGTGCAGCAT	103
	R	CCCGTATTACCGATCCGCTC	
Aldehyde reductase	F	GTGCACCAGATGGAGTGTCA	107
	R	TTCTGGTTACCAAACGGCGA	

Amine oxidase	F	GGTGTCTCCTTCAAGGTCCG	106
	R	GAGTGCGCACATCGGAGATA	
Amino acid permease	F	AGGGCTTTCGTTACTGGAGC	109
	R	CCAGCTTGAATAAGCACCGC	
ATP synthase subunit 6	F	CAGGTTGTCCTTTAGCTTTATTACC	113
	R	GTGACCACTTAATATGTTAGCAGC	
Erg1	F	GAGCAGAAAGTCGGTCCCAA	108
	R	CTCGGTGATACTGGCGTGAA	
Erg3	F	AACGAAGATGGGCGATGAGG	106
	R	CTTCGTGTCGGAGGAGTAGC	
Erg24	F	CAATGGTATGGCAACGGCAG	103
	R	ATGATGGCAGAGGAGAACGC	
Erg25	F	TGGCATTACTTCTCCCACCG	107
	R	GCATATTCAGCAGCCAAGCC	
Carnitine acetyl transferase	F	GGCCATGAGCTCGTATCAGT	104
	R	CGACGATGTGCTGGTTTTCG	
Catalase	F	TTCTGGGATTTCCACGCGAA	106
	R	TACCGAACCCTGTCATTCGG	
Cytochrome b	F	TAATCGACGCACCAACACCA	109
	R	TGCATAGCTAATGTAACTCCTGT	
Cytochrome c oxidase	F	TGGTACTGGTTTCCACGGTT	111
	R	TCGTAACCAAGGTGATGGTGA	
RNA helicase	F	AGGTGAGTTTGCACGAGAGG	101
	R	GAGACGAACGTTCTCCAGCA	
Dienelactone hydrolase	F	TACGGTGATGCTGTGCCTTT	106
	R	CAACGGTTGAGGGGAGATGA	
Epoxide hydrolase	F	CCCACGGCCAAGTATTTCCT	108
	R	CGACCGAACAACCTTCTCCA	

Exopolygalacturonase	F	TGTAAAGCGCATTGGGGGAT	103
	R	TGCTTGCCAGTAATCGGTGT	
Exo-β-1,3-glucanase	F	ATCGATTTTTGACGCCGCTG	103
	R	GCTCCAGTGTTGGGACAGAA	
Fatty acid synthase β subunit	F	CGCAGCATCTACACCACGTA	105
	R	GTAGAGCTTGCTAGCCTCGG	
Fatty acyl-CoA oxidase	F	GTGACTCTCCGGGAACAAGG	107
	R	CCGTGGCCCAATTCTGTTTG	
FMN dependent dehydrogenase	F	GCGGCGCTGATATTGTGAAG	106
	R	TGGCTCACTCCATGTTCACC	
Glutathione S-transferase	F	CTCGGGCTATTGCGAGGTAT	103
	R	CTTGCTGCTTGCTCGAAGAG	
Glycerol kinase	F	ATCAAGGCACTACGAGCACC	102
	R	ACCCCGGGATCGGGTATATT	
Glyoxylate reductase	F	CTTTGTCGTTGCACCGTTGA	107
	R	TTGCCGATGACTCGACTAGC	
Maleylacetoacetate isomerase	F	GCGGCTTGGTGTTGACTTG	101
	R	CCTGTGACCTCCAATGTCCC	
MAP kinase kinase kinase	F	CATTGGATCGCGTGCTTCTG	104
	R	AAGCTCGAGGAAATCAGCCC	
NADH dehydrogenase subunit 1	F	TGCATTTGTAACTATAGCCGAACG	106
	R	AGCATCAGCAAATGCTTGTAAT	
NADH dehydrogenase subunit 4	F	TCTGTATCTCACGCAGCTGTA	108
	R	TGCTGGTGATACAAAACCGT	
NADH-ubiquinone oxidoreductase	F	AGCCCAATCCGATTCAACCA	109
	R	CCTCGTACTGGCACTTCTCG	
Nitrate reductase	F	CCAGATCATGGCCGTCCTTT	102
	R	ATTGCTTGGGGCATCAGTCA	

Nitrite reductase	F	TGCAGGATCGTTGGTCACTC	107
	R	TGCCATCCACATTCTTCCCC	
Oxaloacetate hydrolase	F	ACTGTACGCTGACGGTGTTT	105
	R	AGCGTTGGTCTTCATGTCGT	
Pectate lyase	F	CTCCAAGGCCATTTTGAGCG	104
	F		
	R	CGTTGGTCTCTCCCTTGACC	
Phenyloxazoline synthase	F	GGCTCCATTGATGCCCCATA	106
	R	TCCGGTTTGACGACCCAAAA	
Phosphatidylserine decarboxylase	F	GGTCATCGGGATCTCACAGC	103
	R	CATGATTTGGCGCAGATGGG	
Phospholipase C	F	GACCCTCGGTTACTTGTGGG	108
	R	AGAGTCGGAGGAGTAGAGCG	
Pre-mRNA-splicing factor	F	AAGGAAATCGAAGAGGGCCG	104
	R	CGGTAGTACGGTTCGCTGTT	
Protein-L-isoaspartate O-methyltransferase	F	TTATCACTGGCGATGGACGG	109
	R	CGCAACTGATCCACCAGAGT	
Superoxide dismutase	F	CAGGAAGCGTTTGTCAAGGC	103
	R	TCTCAAGCCGTCCCTTAGGA	
Thioredoxin	F	CATCGCACCCAAGATCGAAG	103
	R	TACACCCAGCTCTTGTCCGA	
Ubiquitin carboxyl-terminal hydrolase	F	ATGGTTCGACAGCTACCACG	101
	R	CACTCCTCGTGCATCTCCTC	
α-L-Rhamnosidase	F	CATGGGTACTGGCAGCAAGA	106
	R	AGACGTGCCATGGTTTGGAT	
β-actin	F	TGTCACCAACTGGGACGATA	101
	R	GAGCTTCGGTCAAGAGGATG	

F and R indicated forward primer and reverse primer, respectively.

Table S2 Summary of the reads quality in the present study.

Sample name	Clean reads	Clean bases	GC Content (%)	Q30 (%)
Pd-d-I	21847213	6536999320	54.06	94.83
Pd-d-NI	23870541	7141474368	53.98	95.00
Pd-wt-I	23294756	6966924166	53.62	94.85
Pd-wt-NI	22149643	6621791146	54.01	94.84

Table S3 Summary of percentages of reads mapping to the reference genome in the present four samples.

Sample name	Pd-d-I	Pd-d-NI	Pd-wt-I	Pd-wt-NI
Total reads	43694426	47741082	46589512	44299286
Mapped reads	41903504 (95.90%)	44888991 (94.03%)	40876823 (87.74%)	41656928 (94.04%)
Unique Mapped reads	40979275 (93.79%)	44103959 (92.38%)	40232931 (86.36%)	40744683 (91.98%)
Multiple mapped reads	924229 (2.12%)	785032 (1.64%)	643892 (1.38%)	912245 (2.06%)
Reads map to '+'	20296757 (46.45%)	21930967 (45.94%)	20120079 (43.19%)	20160549 (45.51%)
Reads map to '−'	20669287 (47.30%)	22221843 (46.55%)	20257660 (43.48%)	20554396 (46.40%)

Table S4 SNP analysis in the present four samples.

Sample name	SNP Number	Genic SNP	Intergenic SNP	Transition	Transversion	Heterozygosity
Pd-d-I	2,458	1,592	866	61.92%	38.08%	33.52%
Pd-d-NI	2,527	1,609	918	61.65%	38.35%	35.30%
Pd-wt-I	2,311	1,511	800	62.48%	37.52%	31.03%
Pd-wt-NI	2,580	1,675	905	61.94%	38.06%	35.47%

Table S5 DEG numbers in the present four comparative groups.

Comparative group	DEG Number		
	All	Up-regulated	Down-regulated
Pd-wt-(I/NI)	460	240	220
Pd-d-(I/NI)	147	50	97
I-(Pd-d/Pd-wt)	1,221	608	613
NI-(Pd-d/Pd-wt)	717	366	351

Table S6 Number summary of the DEGs functionally annotated in the eight public databases.

Database	Pd-wt-(I/NI)	Pd-d-(I/NI)	I-(Pd-d/Pd-wt)	NI-(Pd-d/Pd-wt)
COG	147	32	409	258
KOG	170	35	486	288
GO	243	68	689	408
KEGG	73	18	299	162

Pfam	258	65	750	434
Swiss-Port	196	51	586	346
eggNOG	364	104	1044	577
Nr	426	128	1167	676
Total	427	128	1169	676
